# Polyfunctional HIV-Specific Antibody Responses Are Associated with Spontaneous HIV Control

**DOI:** 10.1371/journal.ppat.1005315

**Published:** 2016-01-08

**Authors:** Margaret E. Ackerman, Anastassia Mikhailova, Eric P. Brown, Karen G. Dowell, Bruce D. Walker, Chris Bailey-Kellogg, Todd J. Suscovich, Galit Alter

**Affiliations:** 1 Thayer School of Engineering, Dartmouth College, Hanover, New Hampshire, United States of America; 2 Ragon Institute of MGH, MIT, and Harvard, Cambridge, Massachusetts, United States of America; 3 Department of Computer Science, Dartmouth College, Hanover, New Hampshire, United States of America; 4 Howard Hughes Medical Institute, Chevy Chase, Maryland, United States of America; Vaccine Research Center, UNITED STATES

## Abstract

Elite controllers (ECs) represent a unique model of a functional cure for HIV-1 infection as these individuals develop HIV-specific immunity able to persistently suppress viremia. Because accumulating evidence suggests that HIV controllers generate antibodies with enhanced capacity to drive antibody-dependent cellular cytotoxicity (ADCC) that may contribute to viral containment, we profiled an array of extra-neutralizing antibody effector functions across HIV-infected populations with varying degrees of viral control to define the characteristics of antibodies associated with spontaneous control. While neither the overall magnitude of antibody titer nor individual effector functions were increased in ECs, a more functionally coordinated innate immune–recruiting response was observed. Specifically, ECs demonstrated polyfunctional humoral immune responses able to coordinately recruit ADCC, other NK functions, monocyte and neutrophil phagocytosis, and complement. This functionally coordinated response was associated with qualitatively superior IgG3/IgG1 responses, whereas HIV-specific IgG2/IgG4 responses, prevalent among viremic subjects, were associated with poorer overall antibody activity. Rather than linking viral control to any single activity, this study highlights the critical nature of functionally coordinated antibodies in HIV control and associates this polyfunctionality with preferential induction of potent antibody subclasses, supporting coordinated antibody activity as a goal in strategies directed at an HIV-1 functional cure.

## Author Summary

A small fraction of HIV-infected subjects mount immune responses that are able to suppress viral replication such that virus cannot be detected in the blood. Understanding how these individuals, known as elite controllers, achieve this outcome may provide a model for strategies to treat or prevent HIV infection. We investigated whether differences in virus-specific antibodies were associated with effective viral suppression. In contrast to other HIV-infected subject groups, antibodies from elite controllers were able to harness the potent anti-viral activities of a wide range of innate immune cells including natural killer cells and phagocytic cells. These effective antibody responses were linked to the presence of different types of antibodies, specifically, functionally potent IgG3 and IgG1 antibodies. Our results indicate that either treatment with or vaccines that are able to drive generation of potent IgG3 and IgG1 antibodies able to recruit the pathogen clearance mechanisms of diverse effector cell types may hold promise in efforts aimed at achieving durable control of viral replication, sometimes described as functional cure of HIV-1 infection.

## Introduction

Vaccine-mediated protection from HIV-1 infection has been observed in humans in association with extra-neutralizing antibody functions, including the ability to induce effector functions such as antibody-dependent cellular cytotoxicity (ADCC) [1]. Similarly, HIV-infected patients who are able to spontaneously suppress infection in the absence of antiretroviral therapy (i.e., HIV-1 controllers) have been found to exhibit potentiated ADCC activity [2–10]. Importantly, as HIV-1 controllers represent an alternative vaccine goal—the induction of immunity able to contain viral replication subsequent to infection—these data suggest that beyond cellular correlates associated with control [11,12], antibodies with enhanced ability to direct the potent anti-viral activities of innate effector cells may also contribute to a functional cure.

Thus, evidence from both protected vaccinees and spontaneous HIV-1 controllers converges on a potential role for non-neutralizing antibody responses with the capacity to direct the cytolytic activity of the innate immune system in viral control and clearance. However, beyond ADCC, antibodies mediate a wide array of additional effector functions, and antibodies from HIV controllers also exhibit elevated phagocytosis, viral inhibition, and NK activation [13,14]. While IgG3-driven antibody polyfunctionality was associated with reduced risk of infection in the RV144 vaccine trial [15,16], the specific humoral profiles that associate with antibody-mediated viral containment in the setting of durable control of infection are unknown.

Accordingly, we aimed to define the functional landscape of anti-viral antibodies among infected subjects with variable degrees of viral control and determine whether specific effector functions, alone or in combination, are associated with durable suppression. Because the functional profile of humoral immune responses may offer insights into both prevention and functional HIV-1 cure, determining the specific characteristics of the most functional anti-viral antibodies offers a unique target for prophylactic and therapeutic HIV vaccines.

## Results

### Controllers do not exhibit enhanced antibody effector functions

**Fig 1:**
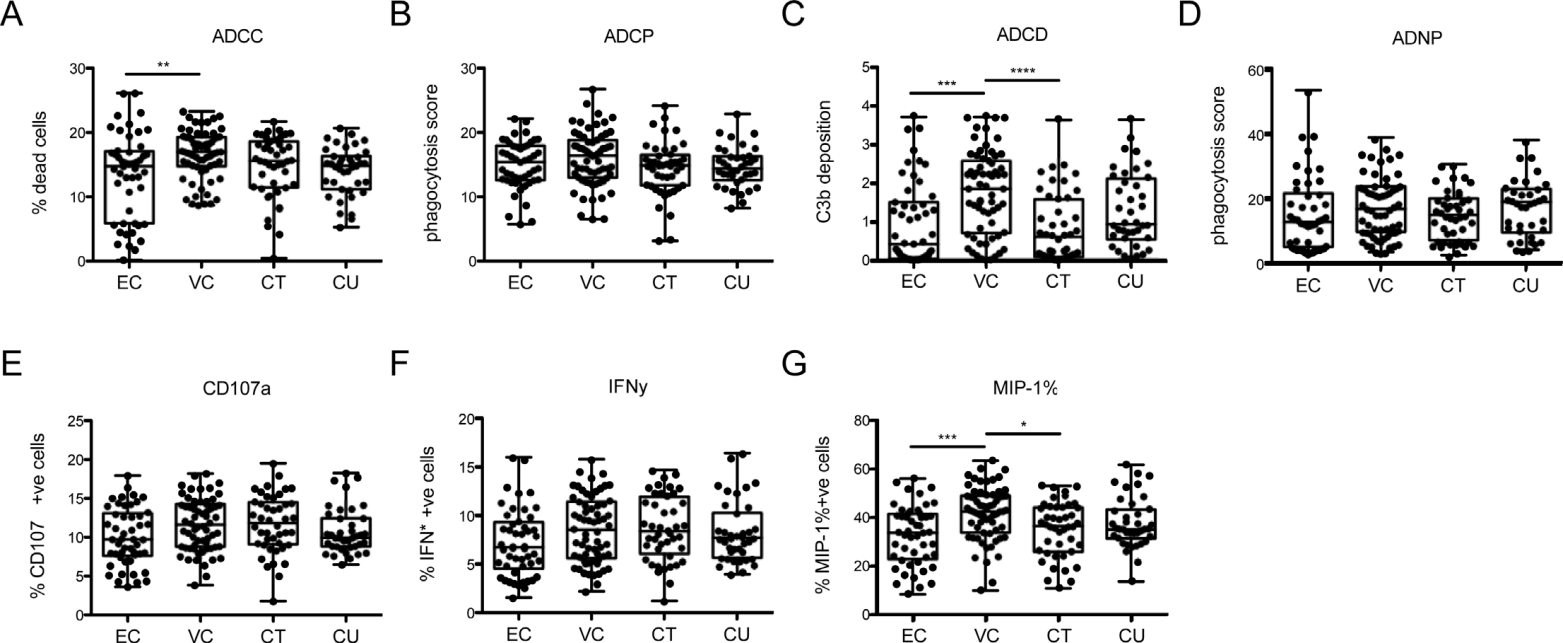
Elite controllers exhibit polyfunctional antibody effector activity profiles in the absence of enhanced antibody responses in individual effector functions. **A-G.** Purified plasma IgG from elite controllers (EC), viremic controllers (VC), infected subjects on therapy (CT), and infected subjects off therapy (CU) were evaluated for their ability to drive NK-dependent ADCC against rgp120-pulsed CD4+ target cells (**A**), monocyte-directed phagocytosis of rgp120-functionalized fluorescent beads (**B**), complement deposition (C3b) on the surface of rgp120-pulsed CD4+ target cells (**C**), neutrophil-directed phagocytosis (**D**), and induce surface expression or production of CD107a (**E**), IFN-γ (**F**), and MIP-1β (**G**) by NK cells in the presence of rgp120-pulsed plates. Differences between subject groups were evaluated using ANOVA adjusted for multiple comparisons using Tukey’s Test in Graphpad Prism.

To broadly profile the functional activity of polyclonal antibodies present in different HIV-positive subject groups, samples from approximately 200 HIV-positive subjects, balanced for sex and age, including elite controllers [12] (EC), viremic controllers (VC), HIV-positive subjects on antiretroviral therapy (CT), and untreated HIV-positive subjects (CU), were evaluated using a suite of functional assays encompassing diverse effector cells and mechanisms. The spectrum of antibody functions evaluated included: (1) antibody-dependent complement deposition (ADCD), which was assessed by measuring the deposition of complement component C3b (derived from HIV-seronegative donor plasma) on the surface of CD4-expressing target cells pulsed with rgp120 [17]; (2) HIV-specific ADCC, which was assessed by measuring CFSE loss from rgp120-pulsed target CEM-NKr cells in the presence of antibody and negatively selected NK cells from healthy donors [16]; (3) antibody-dependent NK cell activation, which was assessed based on the extent of cell surface expression of CD107a on and intracellular production of IFN-γ and MIP-1β in NK cells from healthy donors that had been incubated with antibodies and rgp120-pulsed target CEM-NKr cells [16]; (4) antibody-dependent phagocytosis (ADCP), which was assessed by quantifying the uptake of rgp120-functionalized fluorescent beads by THP1 cells [18,19]; and (5) antibody-dependent neutrophil-mediated phagocytosis (ADNP), which was measured using the ADCP assay method but with primary neutrophils isolated from healthy donors used as effector cells [17]. In contrast to previous studies, enhanced functional activity among ECs was not observed for any individual Fc-effector function compared to VCs, CTs, or CUs (Fig 1A–1G).

### Controllers possess polyfunctional antibody profiles

**Fig 2:**
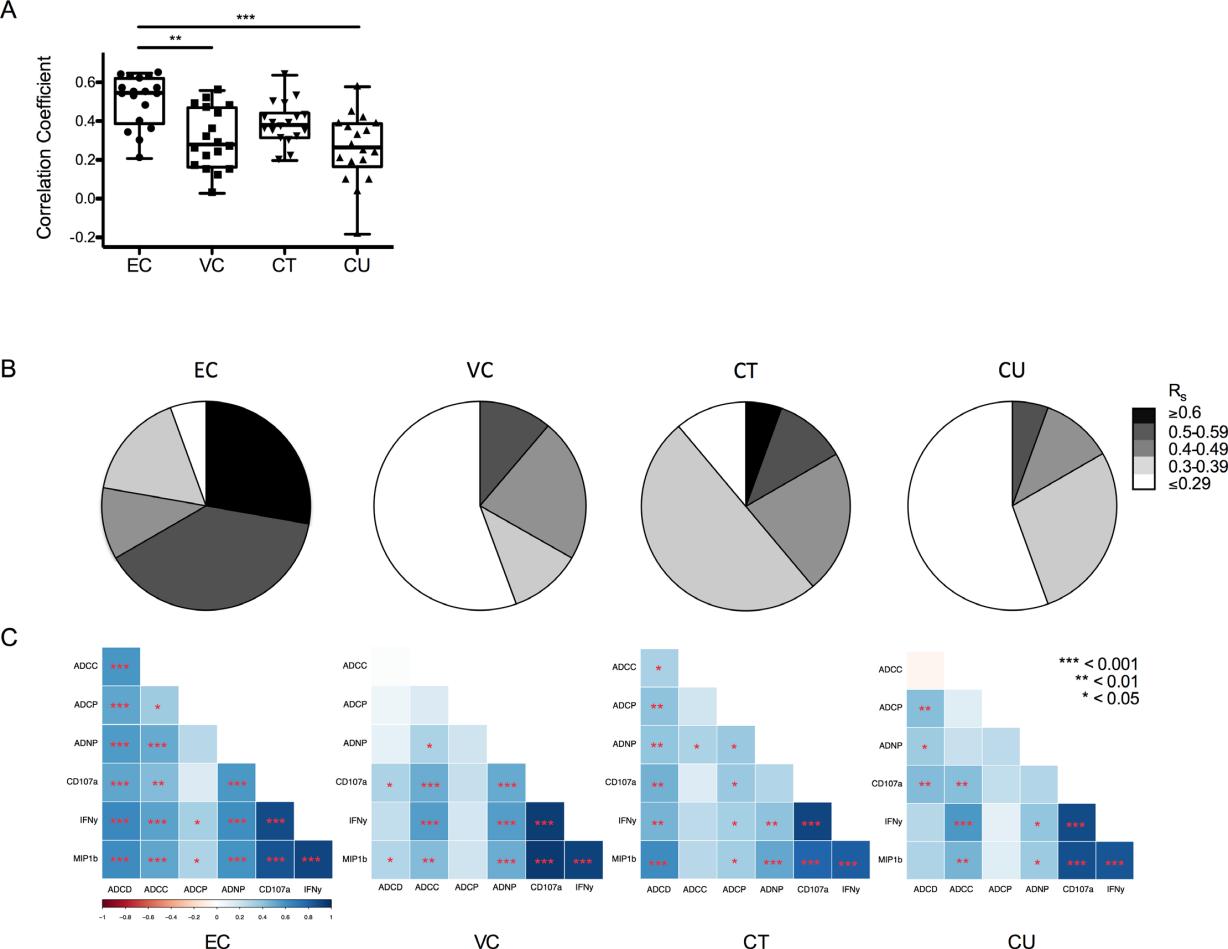
Functional coordination within HIV-infected subject groups. **A.** The extent of functional coordination within groups was assessed by calculating Spearman’s rank correlation coefficients across each pair of independently assessed functional assays. Differences between subject groups were evaluated using a Friedman ANOVA corrected for multiple comparisons using Dunn’s Test in Graphpad Prism. **B**. Prevalence of functional correlations by strength for each subject group. **C**. Correlation matrix for each pairwise combination of functions tested, in which strong positive correlations appear blue while inverse correlations appear red, for each subject group. Correlative relationships and significance were calculated and visualized using R, with unadjusted p values indicated to facilitate relative comparisons.

Because prior analyses of the moderately protective RV144 vaccine trial have suggested that qualitative, rather than quantitative, differences in antibody activity may better mark “protective” responses [15,16,20], we next examined whether there were differences in Fc effector activity coordination. Correlation coefficients of all pairs of effector activities were determined (Fig 2A) and plotted according to their strength and frequency (Fig 2B), demonstrating that antibodies from ECs possessed polyfunctional attributes in the form of a significantly higher degree of functional correlation than antibodies from VCs (*p*<0.01) or CUs (*p*<0.001) and a trend towards higher coordination than CTs. These data demonstrate that even though the magnitude of the effector activity was often individually lower in ECs, the antibody functions were significantly more correlated with one another. Conversely, antibody effector functions were more weakly correlated among the viremic subjects (VCs and CUs) despite generally higher individual antibody Fc-effector activity. Therefore, despite generally lower magnitudes for individual functions, ECs generated a more coordinated antibody effector profile capable of inducing multiple functions simultaneously. Furthermore, when broken out across each pair of functions, these correlation analyses identified unique profiles within the subject groups (Fig 2C). For example, ADCD was highly positively correlated with all other functions in ECs, but generally had no or only weak correlation with the other functions, particularly in the viremic subject groups (VC and CU). Overall, the differences in functional coordination observed between subject groups further support observations from the RV144 analysis [15,16,20] and NHP studies [17,21], demonstrating that qualitatively superior responses may provide better antiviral defense.

### Levels and IgG subclass composition differentiate HIV+ subject groups

**Fig 3:**
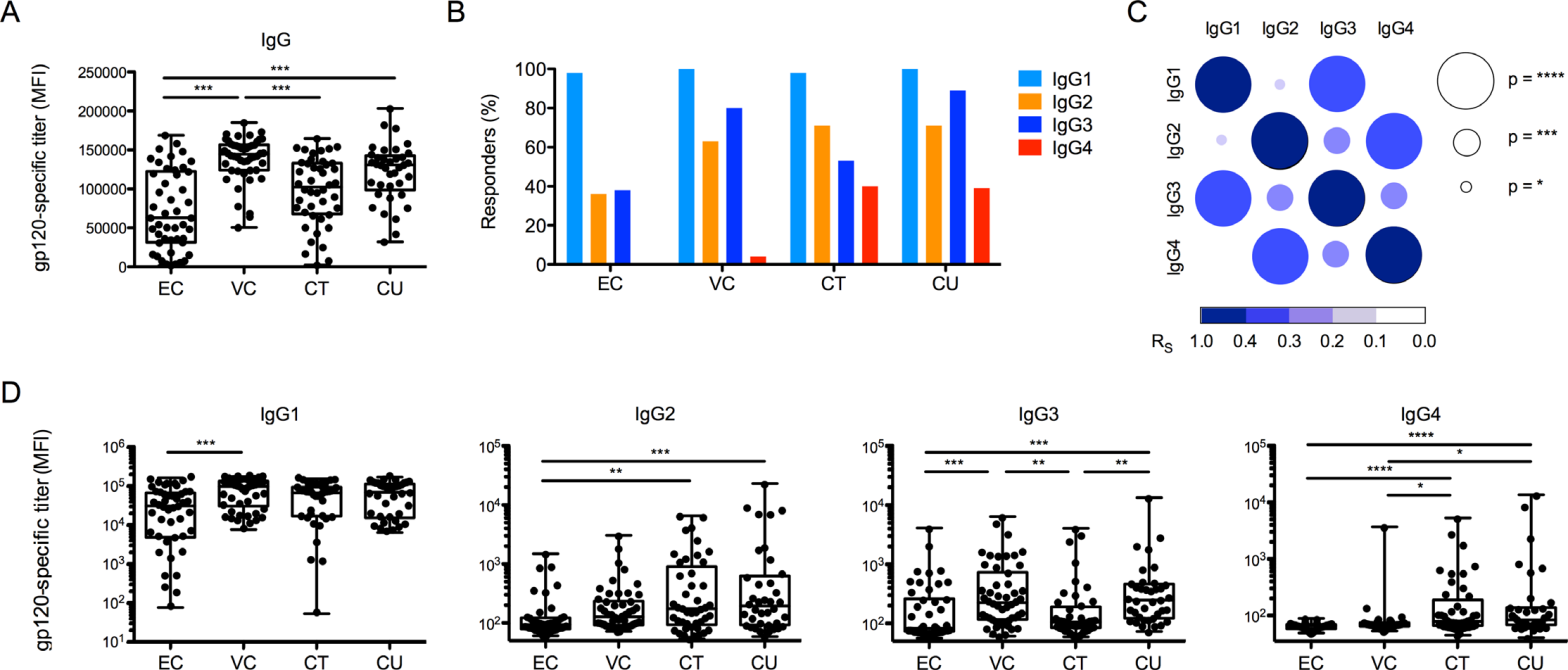
Levels and IgG subclass composition differentiate HIV+ subject groups. **A.** Titer (Mean Fluorescent Intensity, MFI) of gp120-specific IgG present in each subject group. **B**. The percent of subjects in each group positive for gp120-specific responses of each IgG subclass. **C.** Spearman correlation matrix between subclass responses across subjects. Strength and significance, as calculated in Graphpad Prism, are represented as color intensity and size, respectively. **D.** The levels of gp120-specific responses observed across cohort groups for each IgG subclass. Differences between groups were assessed by Kruskal-Wallis ANOVA and corrected for multiple comparisons using Dunn’s test in Graphpad Prism.

Antibodies were titered in order to begin to dissect the level and types of antibodies associated with potent and coordinated effector function (Fig 3A). Viremic subject groups (VC and CU) possessed significantly higher antibody responses than ECs, with a median level that was approximately twice as great as the level observed in ECs. The differences between the various groups were larger when titer, rather than function, was examined, suggesting that on a per-molecule basis, antibodies from ECs may be more functionally potent than those present in other subject groups.

Because mechanistic studies aimed at dissecting the underlying biology of polyfunctional antibody responses in RV144 pointed to a critical role for the induction of HIV-specific IgG3 antibodies [15,16,20] and negative impacts of the induction of IgG2, IgG4, and IgA antibodies [1,20,22], we aimed to move beyond determination of total IgG levels and examined IgG subclass selection across the cohort. The percent of subjects who elicited antibodies of each subclass was determined (Fig 3B), and while gp120-specific IgG4 responses were more prevalent in the CTs and CUs, IgG4 responses were virtually absent in the controllers (VC and EC). Most VC, CT, and CU subjects generated an IgG2 response, whereas the majority of ECs did not generate gp120-specific IgG2 antibodies. While the IgG3 responses varied among the groups, these responses were generally more prevalent in the viremic subjects.

### Subclasses with corresponding activity profiles tend to be co-induced

Because IgG subclasses possess distinct functional profiles, with IgG3/1 demonstrating strong binding to FcγRs and C1q but IgG2/4 exhibiting weak binding and effector activity, we wondered whether coordination among the subclasses, representing skewing towards synchronously more or less active subclasses, could be observed in the study cohort. When the degree of correlation between response levels for each subclass was determined, we observed that subclasses with similar activity profiles (i.e., IgG1/IgG3 or IgG2/IgG4) had strong positive associations, whereas only weak or no associations were observed between subclasses with opposing activity profiles (e.g., IgG1/IgG4; Fig 3C). This observation suggests that the humoral immune system may integrate signals to define antibody activity at a relatively high level, predisposing B cells to class-switch to either IgG3/1 or IgG2/4 based on the level of antibody effector potency desired.

This type of activity-based subclass skewing could be observed within groups, where notable differences in subclass response magnitudes were also observed (Fig 3D): relatively elevated levels of gp120-specific IgG3/1 responses were observed among controllers, whereas higher levels of gp120-specific IgG2/4 responses were apparent in chronically infected subjects, particularly compared to ECs. These data highlight significant subclass selection biases between controllers/non-controllers, especially the induction of subclasses with compromised function among subjects with progressive disease.

### Polyfunctionality is associated with differential subclass skewing in Controllers

**Fig 4:**
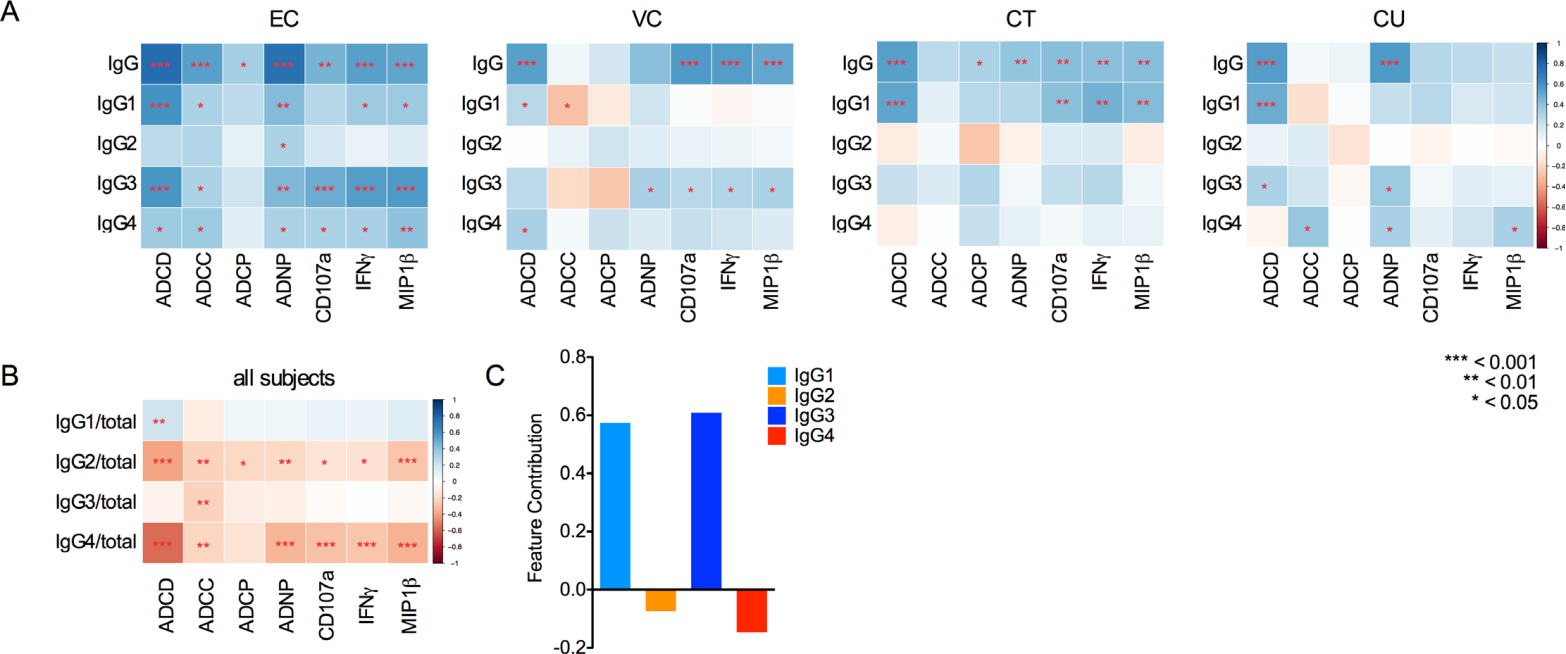
Antibody functionality can be predicted by subclass composition. **A**. Correlative relationships between levels of total gp120-specific IgG or gp120-specific antibodies of each subclass to antibody function were assessed by determination of Spearman’s rank correlation coefficients between activity and antibody MFI within each subject group. **B**. Correlative relationships between *relative* levels of gp120-specific antibodies of each subclass (i.e., MFI of subclass/MFI of total IgG) were assessed for each functional activity, over all subjects. Positive associations are noted in blue and inverse associations in red, with the magnitude of correlation depicted by intensity. Correlative relationships and significance were calculated and visualized using R, with unadjusted p values indicated to facilitate relative comparisons. **C**. The magnitude and direction of the contribution of SF162-specific antibody subclass assessments to cross-validated predictive models of polyfunctional activity.

We next aimed to define the relationship between antibody subclass levels and functionality for each subject group (Fig 4A). Total HIV-specific antibody levels correlated with every antibody function in ECs and nearly all functions in CTs; however, total HIV-specific antibody levels in VCs and CUs failed to correlate with most functions tested. These data suggest that the “average” antibody present in ECs is functional, whereas the observation that titer does not relate to activity in VCs and CUs indicates that there are striking differences in the overall quality of antibody pools present among groups.

Deconvolution of the relationships of individual subclasses with effector functions revealed strong relationships between HIV-specific IgG3 subclass levels and nearly all effector functions uniquely among the ECs. Correlations observed for IgG1 likewise suggest that beyond IgG3, qualitatively superior IgG1 antibodies bearing the capacity to drive polyfunctional antibody responses were also induced. In some cases, weakly negative relationships were observed between IgG subclass responses and effector function, further demonstrating that higher antibody titers do not necessarily drive enhanced functionality and that qualitative differences in subclass profiles may play a critical role in driving more effective antibody activity and achieving potent viral suppression.

Based on this observation, and because the formation of immune complexes is required to trigger Fc-effector function, we examined the effect of subclass ratios on antibody functionality. Independent of overall titer, elevated relative levels of IgG2 and IgG4 were strongly negatively associated with antibody functionality across all subjects (Fig 4B), supporting previous observations of the potentially inhibitory activity of these less-functional subclasses [16,20] and further illustrating the critical nature of qualitative antibody differences in driving a broader array of Fc-effector functions that may enable more robust control of viral replication.

### Modeling potent polyfunctional activity

To begin to establish whether consistent relationships between subclass and IgG activity could be identified when overall response level and subclass distribution were considered across all subject groups, we built models of polyfunctional activity using a generalized linear model machine learning method [23]. In this effort, the subclass response data was used to train a classifier to discriminate between subjects with either poly- or mono-/non-functional antibody responses in the setting of fivefold cross-validation. The resulting models predicted polyfunctional class significantly better than when class assignments were permuted (balanced accuracy = 0.65 ±0.02 vs. permuted balanced accuracy = 0.49 ±0.02; *p*<1x10^-15^) and, thus, reliably capture the quantitative impact of subclass distribution and levels on antibody polyfunctionality. Consistent with a previous study of RV144-induced antibodies [20], IgG3/1 responses made strong positive contributions to the models, whereas IgG2/4 responses were associated with reduced activity in models of antibody polyfunctionality (Fig 4C). Importantly, despite the relatively lower levels of IgG2/3/4 responses as compared to IgG1, these subclasses made strong contributions to polyfunctional classification. These results are consistent with the finding that IgG4 can negatively impact activity and that IgG3 can positively impact activity, despite low titer, which were experimentally confirmed in polyclonal samples from RV144 by subclass depletion experiments [16]. Collectively, these models further support the importance of antibody quality and subclass composition in defining the functional profile of polyclonal sera.

## Discussion

While the development of an HIV vaccine able to induce neutralizing antibodies that block HIV acquisition represents the ultimate goal in HIV vaccine design, vaccines to date have had limited success in inducing these types of immune responses. Conversely, protection from infection has been observed in both humans [1] and non-human primates [17] in the absence of potent neutralization. Instead, non-neutralizing antibody mechanisms, including the ability of antibodies to induce antibody effector functions, have been implicated in protective activity.

ECs represent a unique model of functional cure in that these individuals have spontaneously developed HIV-specific immunity that is able to persistently contain the viral reservoir to undetectable levels. While cellular immunity has been implicated in this control [11], other arms of the immune response, including functional antibodies, have also been observed in these individuals. Here, by probing an array of distinct innate immune effector mechanisms, we show for the first time that while neither the overall magnitude of antibody function nor titer is higher in ECs, these individuals uniquely induce a more coordinated innate immune–recruiting antibody response. Specifically, while ECs demonstrate individual antibody effector functions comparable to other groups, these activities are evoked in the setting of a lower antibody titer. Additionally, these functions are coordinated within the humoral immune response in such a way that individuals who induce antibodies with the capacity to recruit ADCC also generate antibodies able to drive phagocytosis by both monocytes and neutrophils, induce complement deposition, and stimulate NK cell degranulation and cytokine/chemokine secretion. Whether these functions are induced by the same antibodies or through a network of highly coordinated functional B cells is unclear, but these antibodies represent a humoral immune response that may broadly direct the antiviral capacity of any innate immune cells found within a tissue compartment where the reservoir may persist. Importantly, this superior humoral immune response was associated with the overall levels of HIV-specific IgG3 and IgG1, highlighting the critical nature of qualitative differences in directing potent humoral immunity against HIV.

By contrast, there were no or only weak relationships observed between antibody titer and functional activity in some subject classes, notably VCs and CU subjects, as has been previously observed [24]. Interestingly, coordinated humoral immunity was also lost in the setting of viremia. In combination, these observations indicate a dysfunctional response that, in correlative and predictive machine learning models, was quantitatively associated with IgG2/4 skewing. Consistent with the results of structured treatment interruption, which generally but not universally results in rapid viral rebound [25–27], functional coordination appeared only partially restored in the setting of antiretroviral therapy (i.e., in CTs). However, this restoration of function was not associated with an improved relationship between antibody Fc-effector functions and IgG3 levels. Instead, this function was associated with the levels of IgG1 antibodies directed at the viral envelope. These data suggest that, in the setting of reduced viremia, qualitatively superior IgG1 antibodies may be induced in CTs that may display polyfunctional activity similar to the IgG3 antibodies in ECs, potentially through the selective glycosylation of these IgG1 antibodies [14] to enable higher affinity interactions with a broader array of Fc receptors. Because IgG1 levels also correlate with Fc-effector functions in ECs, albeit to a lower level than IgG3 responses, it is plausible that similar highly functionalized IgG1 antibodies may be induced in parallel to IgG3 antibodies and that these potent IgG1 antibodies collaborate in the induction of polyfunctional antibody profiles in ECs. Importantly, these results point to two possible features key to highly functional antiviral immunity targeting the viral reservoir with relevance to the design of next-generation therapeutic vaccines and passive transfer strategies for functional cure: (1) the production of high levels of IgG3 responses and (2) the targeted induction of highly functional, uniquely glycosylated IgG1 antibodies.

It will be important to both confirm and potentially extend these observations. Previous work has yielded conflicting results as to whether antibody effector activity may stratify by the presence or absence of protective HLA-B alleles [28,29], resolution on this and evaluation of other subsets of controllers could provide refined understanding with high relevance to functional cure and vaccine efforts. Among controllers, three levels of suppression have been identified, including: 1) viremic controllers, able to control viremia at detectable levels 50–2000 copies/ml; 2) elite controllers, able to control viremia to undetectable levels (<50 copies/ml); and 3) super-elite controllers, able to control viremia to undetectable levels by the most sensitive detection methods. While we observed distinct antibody Fc-effector profiles among viremic and elite controllers, a third profile may exist among the super-elite that could represent the ultimate example of humoral functional cure. Thus, future efforts aimed at defining both unique profiles of individual monoclonal responses within super-elite controllers, as well as the overall functionality of polyclonal humoral responses associated with robust pressure on the viral reservoir, are warranted in these subjects who suppress virus to extraordinarily low levels.

Moreover, evaluation of antibody functionality in the setting of post-treatment control, an occasionally observed phenomenon in which viral suppression is maintained following treatment interruption, could elucidate the specific antibody functions and targets that may most effectively control or eradicate the reactivatable reservoir. While our study was not designed to specifically profile humoral immunity in the setting of either super-elite or post-treatment control, we observed markedly enhanced antibody functionality in subjects on treatment relative to those off-therapy, suggesting the possibility that the humoral immune response may improve over years of therapy. That these responses may evolve longitudinally suggests that it may be possible to enhance them by therapeutic vaccination, to more aggressively promote post-treatment control. In this regard, observations linking early treatment with post-treatment control [30–32] are particularly interesting given the rapid decay observed in antibody function and IgG3 levels during acute infection in the absence of therapy [33,34].

Interestingly, the protection from infection in the moderately protective RV144 vaccine trial was associated with an enrichment of polyfunctional IgG3 responses in subjects who resisted HIV infection [15,16]. Combined with the profiles observed in ECs in the current study, these data argue that vaccines able to induce IgG3 responses prior to infection may also have the capacity to potentially provide protection from infection. Importantly, because IgG3 is the first subclass in the immunoglobulin locus, vaccine-induced immunity must drive class switch recombination to IgG3 and prevent further downstream switching to IgG1 or other less functional antibody subclasses. Promisingly, tetanus vaccination drives IgG3 antibody responses [35], and repeated tetanus boosting specifically promotes IgG3 recall responses [35], suggesting that the selective induction of IgG3 antibodies is possible. However, little is known about the specific mechanism by which IgG3 responses are selected and locked into memory. The apparently coordinated regulation of subclass switching implies that the signals integrated by B cells may result in coordinated function and that the identification of the signals responsible for alternatively potent or weak effector responses may hold promise across vaccines aiming for potent pathogen clearance or tolerizing responses to autoantigens or allergens. Future HIV vaccine studies aimed at defining the mechanism by which functional IgG3 responses may be selectively induced via vaccination may offer a unique opportunity to test whether HIV-specific IgG3 antibodies are able to provide protection from infection or post-infection control and clearance of the viral reservoir.

While we have studied the impact of subclass selection primarily on non-neutralizing antibody effector function, several neutralizing antibodies were originally cloned as IgG3, and it is important to note that previous studies have linked this subclass to potentiated neutralization activity in polyclonal responses [36], as well as in the setting of monoclonal antibodies [37–39]. Importantly, the neutralization enhancements observed for subclass-switched mAbs imply that beyond the well-known impact of subclass and glycosylation on FcγR binding and effector function, structural differences between subclasses and glycovariants can impact other potentially protective antibody activities. Toward this end, the most intriguing evidence for the impact of such structural factors can be found in the superior neutralization activity of polyclonal, serum-derived bivalent IgG3 Fab’_2_ fragments relative to IgG1 Fab’_2_, whereas equivalent neutralization potency was observed for monovalent IgG1 and IgG3 Fab fragments [36]. While previous study of the neutralization capacity of our study cohort did not indicate significant differences between groups [14], the links between neutralization potency and subclass in the setting of monoclonal antibodies may be highly relevant for mAb therapy.

In summary, the polyfunctional IgG1/3 responses identified here as associated with variable viral suppression may also be relevant to therapeutic and/or prophylactic antibody strategies [40,41], whether by novel vectored strategies or by traditional passive transfer. That passively acquired, functionally competent antibodies may be protective in the setting of human transmission has been previously observed and recently confirmed in the setting of mother-to-child transmission [42,43]. Collectively, as both antibody-based therapy and prevention strategies advance, our data from the setting of elite viral control expands existing data from protective human and NHP vaccines and passive immunity in infants by pointing to the high potential relevance of maximizing antibody effector function breadth and potency.

### Conclusion

Overall, despite lower activity and titer, coordinated humoral immunity was uniquely present among ECs and associated with virus-specific IgG3/1 responses and the absence of IgG2/4 responses. Functional coordination was lost in the setting of viremia, but appeared to be partially restored by antiretroviral therapy in association with the recuperation of enhanced IgG1 functionality. While it is unclear whether these functions are induced by the same antibodies or through a network of functionally tuned B cells, these data suggest that qualitatively superior IgG3/1 antibodies may collaborate to broadly direct the antiviral capacity of diverse local innate immune cells to suppress viremia. This study provides critical insights into the features of highly functional IgG3/1-biased antiviral immunity that may contribute to durable control of the HIV reservoir, whether achieved by vaccination or passive transfer. In doing so, it reinforces findings from effective human [1,15,16] and NHP [17,21] vaccines regarding the potential significance of harnessing polyfunctional antibodies to prevent infection and thus highlights the contribution that polyfunctional antibody responses may make to both HIV-1 prevention and therapy.

## Materials and Methods

### Subjects

187 HIV-positive subjects, balanced for sex and age, were analyzed for this study, including 50 Elite Controllers [12] (<50 copies RNA/ml, EC), 64 Viremic Controllers (50–2000 copies of RNA/ml, VC), 45 HIV-positive patients on antiretroviral therapy (ART) (<50 copies RNA/ml, Chronic Treated, CT), and 38 untreated HIV-positive patients (>50 copies RNA/ml, Chronic Untreated, CU). Controllers were chosen from a cohort of HIV-1-infected individuals that has been described previously[12]. EC were defined as subjects with plasma HIV RNA levels <50 or <75 copies/ml based on a minimum of 3 determinations of plasma HIV RNA spanning at least a 12-month period in the absence of anti-retroviral therapy. IgG was purified from plasma samples using the Melon Gel IgG Spin Purification Kit (Thermo Scientific).

### Ethics statement

The study was reviewed and approved by the Massachusetts General Hospital Institutional Review Board, and each subject gave written informed consent.

### Antibody-dependent cellular phagocytosis (ADCP)

HIV-specific phagocytic activity was assessed using a flow cytometry-based phagocytic assay as described previously [18,19]. Briefly, fluorescent, streptavidin-microspheres were coated with biotinylated gp120 SF162 protein (Immune Technology), and the ability of purified IgG antibodies to drive uptake by the monocytic THP-1 cells was assessed by flow cytometry.

### Antibody-dependent complement deposition (ADCD)

Antibody-dependent complement deposition was assessed by measurement of complement component C3b on the surface of target cells [17]. CD4-expressing target cells were pulsed with gp120 SF162 (60 mg/ml), and incubated with antibodies. Purified IgG was combined with pulsed or unpulsed target cells and freshly harvested HIV negative donor plasma diluted with veronal buffer 0.1% gelatin (1:10 dilution) to allow for complement deposition. Replicates using heat inactivated donor plasma were used as negative controls. Cells were incubated for 20 min at 37°C, then washed with 15 mM EDTA in PBS. Complement deposition was detected by staining for C3b-FITC (Cedarlane). Cells were fixed and the proportion of target cells with C3b-FITC deposition was analyzed by flow cytometry.

### Antibody-dependent cellular cytotoxicity (ADCC)

An adaptation of the rapid fluorometric ADCC (RFADCC) assay[44] was used to assess NK-cell mediated target cell killing. In brief, the CEM-NKr T lymphoblast cell line was labeled with the intracellular dye carboxyfluorescein diacetate succinimidyl ester CFSE and the membrane dye PKH26 and then pulsed with gp120 SF162 protein (60 μg/ml). NK cells were enriched directly from healthy donor whole blood by negative selection using RosetteSep (Stem Cell Technologies). Purified IgG were added to the labeled CEM-NKr cells and incubated with NK cells for 4 hr at 37°C. The 1:5 target to effector cell mix was fixed, and the proportion of lysed cells (those that maintained membrane expression of PKH26 but had lost intracellular CFSE) was determined by flow cytometry.

### Antibody-dependent NK cell activation

An assay to determine the expression of surface CD107a and intracellular production of IFN-γ and MIP-1β was performed by pulsing the CEM-NKr CCR5+ T lymphoblast cell line with gp120 SF162 (60 μg/ml), as previously described [16]. NK cells were isolated from whole blood from healthy donors using negative selection with RosetteSep (STEMCELL Technologies), then combined with CEM-NKr cells at a ratio of 5:1. Purified IgG, anti–CD107a–phycoerythrin (PE)–Cy5 (BD), brefeldin A (10 mg/ml) (Sigma), and GolgiStop (BD) were added for 5 hours at 37°C. The cells were then first stained for surface markers using anti–CD16–allophycocyanin (APC)–Cy7 (BD), anti–CD56-PE-Cy7 (BD), and anti–CD3–Alexa Fluor 700 (BD) and then stained intracellularly with anti–IFN-γ–APC (BD) and anti–MIP-1β–PE (BD) using Fix and Perm A and B solutions (Invitrogen). The cells were then fixed in 4% paraformaldehyde and analyzed by flow cytometry. NK cells were defined as CD3-negative and CD16- and/or CD56-positive.

### HIV-specific antibody subclassing

A customized multivariate multiplex assay was utilized to characterize the subclass of HIV-specific antibodies, as previously described [45]. Responses against HIV gp120 from SF162 are reported since this was the specific antigen utilized in functional assessments. HIV-IG (NIH AIDS Reagent Program) and pooled HuIgG from HIV negative donors (Sigma) were used as positive and negative controls.

### Statistical analyses

Comparative analyses of antibody properties between subject groups were performed by ANOVA in GraphPad Prism with corrections for multiple comparisons applied as described in figure legends. Spearman correlation coefficients were calculated with a two-tailed p values with 95% confidence intervals in either GraphPad Prism or using the R software package[46], version 3.1.2, again, with statistical tests applied as described in figure legends.

Predictive models of polyfunctional activity were built using the Glmnet R package for generalized linear models [23]. Polyfunctional activity was determined by first discretizing functional assay measurements into high/low activity using a median split, then counting the number of assays in which each subject had high activity. Subjects with high functional activity in two or more functions were classified as polyfunctional, and those high in one or none, as non-polyfunctional. A classification model was trained using antibody subclass profile data at a Ridge regression elastic net setting of zero, and used to determine the coefficient weight of each IgG subclass assessment contributing to the model. Predictive accuracy and robustness was assessed with 250 iterations of 5-fold cross validation, and compared to analogous results from 250 iterations of 5-fold cross validation with polyfunctional class labels randomly permuted.
